# Supplementary material for: Massive methane fluxing from magma–sediment interaction in the end-Triassic Central Atlantic Magmatic Province
Source: Nat Commun. 2021 Sep 20;12:5534. doi: 10.1038/s41467-021-25510-w (PMC8452664; doi:10.1038/s41467-021-25510-w)
Supplement: Supplementary file 1 — Description of Additional Supplementary Files [file 41467_2021_25510_MOESM1_ESM.pdf]

Supplementary Information

Massive methane fluxing from magma-sediment interaction in the end-Triassic Central Atlantic Magmatic Province

Capriolo et al.

Supplementary Notes

Supplementary Note 1. All the rock samples containing quartz display FIs within this mineral phase. The 10 most representative quartz-bearing samples were characterized by optical microscopy and by XRF spectrometry, in terms of major element composition (Supplementary Data 1, 2). The investigated samples belong to both low-Ti (<2.0 wt.% TiO_2) and high-Ti (>2.0 wt.% TiO_2) groups. These rock samples are gabbroic in composition, with holocrystalline, hypidiomorphic texture and fine to medium grain size, typical of shallow intrusions, and are thus classified as microgabbros in this study (Figs. 2, 3; Supplementary Figs. 2, 3). An evident disequilibrium between anhydrous mineral phases (crystallized during the early-magmatic stage) and hydrous mineral phases (crystallized during the late-magmatic stage) is revealed by the common substitution of early minerals. All analysed microgabbros show variable degrees of hydrothermal alteration (likely due to the circulation of hydrothermal fluids) at the expense of both early- and (less commonly) late-magmatic minerals.

Early-magmatic minerals are calcic plagioclase, olivine (in quartz-free samples only), clinopyroxenes (mainly augite and pigeonite) and oxides (ilmenite and magnetite). Both oxides often display a skeletal crystal habit. Within unaltered clinopyroxene crystals, bubble-bearing melt inclusions, similar to those containing CO_2 mainly found in CAMP lava flows[1], are rarely preserved. Late-magmatic minerals are sodic plagioclase, brown hornblende, red biotite, quartz, alkali feldspar, apatite, zircon, oxides (ilmenite and magnetite) and sulfide (pyrite). Brown hornblende, red biotite and (less commonly) oxides locally replace the early-magmatic clinopyroxenes, instead pyrite often replaces both early-magmatic oxides. Hydrothermal minerals are sericite, actinolite, chlorite, green biotite, epidote, carbonate, quartz and sulfides (chalcopyrite and pyrrhotite), and occasionally form hydrothermal veins crosscutting microgabbros (sample RP128; Supplementary Fig. 4).

Supplementary Note 2. Quartz, which is the main host mineral phase for FIs, occupies an interstitial position in the microgabbros and represents on average 5 vol.% of the whole rock (up to ca. 13 vol.% in some samples), consistently with CIPW normative compositions calculated from whole-rock chemical compositions (Supplementary Data 1, 2). Quartz is present as single crystals or aggregates, with eu- to an-hedral crystal habit, or in irregular graphic textures intergrown with alkali feldspar, and is often associated with opaque mineral phases, especially with pyrite, and sometimes with ilmenite and/or chalcopyrite. Moreover, quartz is also present within hydrothermal veins in crystals

different from late-magmatic quartz crystals for their higher transparency, mainly due to the minor presence of inclusions.

Primary FIs (i.e., entrapped during host mineral crystallization) show a random distribution within quartz crystals, range approximately from 1 to 50 µm as maximum dimension and generally contain 2 or 3 phases (liquid + vapour ± solid phases) at ambient temperature, resulting liquid- or vapour-rich usually in association with each other. The liquid phase is always transparent and colourless, whereas the vapour phase is usually transparent and smoke grey, characterized by a thick boundary with evident refractive effects (i.e., the vapour bubbles show an external green rim and an internal red rim by optical microscopy). Primary FIs are also locally present in feldspars crystallized together with quartz, within the microgabbro, and in hydrothermal quartz and calcite, within the hydrothermal vein.

Secondary FIs (i.e., entrapped after host mineral crystallization) are usually vapour-rich and are similar to primary FIs, but show a distribution along healed fracture planes and a common negative crystal shape. These FIs are present in quartz, plagioclase and calcite crystals, but were not further investigated as they may have been entrapped well after the formation of magmatic and hydrothermal crystals.

Primary liquid-rich FIs (i.e., containing liquid + vapour ± solid phases) in late-magmatic quartz are colourless, generally with irregular shape and sharp contour. The liquid phase occupies most of the volume within the inclusion, the vapour phase forms a spherical bubble and one or more transparent solid phases (often displaying a cubic shape) can be present. When more solid phases are present within the same liquid-rich FI, their shape and optical properties (e.g., colour) are often different, highlighting their different nature. In liquid-rich FIs, the vapour phase generally occupies from 5 to 25 vol.%, exceptionally up to 50 vol.% of the whole FI.

Primary vapour-rich FIs (i.e., containing vapour ± liquid phases) in late-magmatic quartz are smoke grey, generally with elongated shape and sharp contour. The vapour phase occupies most of the volume within the inclusion and the liquid phase, which is not always present, forms thin films along boundaries and in corners of the inclusion as liquid menisci. In vapour-rich FIs, the vapour phase generally occupies from 75 to 100 vol.%, averaging about 95 vol.% of the whole FI.

Microthermometry of liquid-rich FIs was carried out on 4 samples (Supplementary Data 1). Last-melting temperatures at 0 °C, measured on 2 phase-bearing FIs (two FIs in sample RP134), indicate 0 wt.% salinity. Homogenization temperatures (always occurring in liquid phase for the analysed FIs) between 150 and 304 °C, measured on 2 and 3 phase-bearing FIs (two FIs in sample RP106, one FI in sample RP116, two FIs in sample RP128 and four FIs in sample RP134), constrain the minimum temperature for FI entrapment.

Confocal Raman microspectroscopy analyses on FIs, hosted in late-magmatic and hydrothermal quartz and marginally in hydrothermal calcite, were carried out on the 10 most representative samples (Supplementary Data 1, 3; Source Data 1). Hyperspectral Raman maps allowed to reconstruct and image the spatial distribution of liquid and vapour phases, but were acquired only for vapour-rich FIs since moving vapour bubbles hindered the areal analysis of liquid-rich FIs.

In the FIs hosted in late-magmatic quartz, the liquid phase is H_2O, the vapour phase is CH_4 and the main solid phase is NaCl (halite), for all analysed samples (Figs. 3, 4). The lack of other fluid phases (N_2, H_2, H_2S, SO_2, CO, CO_2 and CH_3Cl) was checked by confocal Raman microspectroscopy within randomly selected FIs hosted in late-magmatic quartz. In the FIs hosted in hydrothermal quartz within hydrothermal vein (sample RP128), both liquid and vapour phases are H_2O and the main solid phase is possibly NaCl. In the FIs hosted in hydrothermal calcite within hydrothermal vein (sample RP128), the liquid phase is H_2O, the vapour phase is CO_2 and the main solid phase is possibly NaCl.

Liquid H_2O is characterized by a broad Raman band in the range ca. 3000-3700 cm^{-1} (ref. 2). Gaseous CH_4 is characterized by an intense, sharp Raman band at ca. 2917 cm^{-1} and by a weak Raman band at ca. 3022 cm^{-1} (refs. 2, 3). Moreover, CH_4 is often partially dissolved within liquid H_2O, as revealed by a weak Raman band at ca. 2910 cm^{-1}, close to the main Raman band of gaseous CH_4[3-5]. Because of the resonance effect, gaseous CO_2 is characterized by two sharp Raman bands (Fermi diad or Fermi doublet), at ca. 1285 and 1388 cm^{-1}, associated with two symmetrical weak Raman bands (hot bands), below 1285 cm^{-1} and above 1388 cm^{-1} (refs. 2, 6). Solid NaCl is not Raman-active, thus it was detected by confocal Raman microspectroscopy combined with microthermometry within some FIs hosted in late-magmatic quartz (samples RP106, RP116 and RP128). Cooling down a system containing NaCl and H_2O converts halite into hydrohalite, which is Raman-active.

The density of gaseous CH_4 was calculated through the position of the main Raman band of CH_4 calibrated with neon emission lines (Supplementary Data 3). We used the densimetry calibrations of references 7 and 8, which are optimal for low densities. In detail, after correcting the position of the main Raman band of CH_4 according to equation 1 of reference 9, modified for the position of neon emission lines at 2836.976 and 2933.916 cm^{-1}, we used equation 5 of reference 7 and equation 2 of reference 8. The measured density of gaseous CH_4 is similar within both liquid- and vapour-rich FIs and generally ranges from 0.01 to 0.04 g/cm^3 (exceptionally up to 0.17 g/cm^3 for one vapour-rich FI in sample RP116), averaging about 0.02 g/cm^3.

The salinity in liquid-rich FIs is strongly variable. Even within single quartz crystals, halite-bearing and halite-free FIs coexist. Aside from the marginal presence of CH_4 within the liquid-rich FIs, the occurrence of NaCl as crystals in the liquid H_2O at ambient temperature indicates >26 wt.% salinity, corresponding to the peritectic composition[10]. Some FIs display

0 wt.% salinity, as revealed by microthermometry and confirmed by Raman spectral features of liquid H_2O, thanks to the systematic variation of the shape of its Raman band with salinity[6]. The liquid H_2O menisci of vapour-rich FIs display ca. 10 wt.% salinity, as estimated for the Raman mapped FIs via their Raman spectral features[6].

Confocal Raman microspectroscopy combined with microthermometry on FIs was carried out on 4 samples (Supplementary Data 1; Source Data 2). In the analysed FIs, the phases forming from the H_2O-NaCl-CH_4 system between -190 and 0 °C can be water ice, methane clathrate and hydrohalite (Fig. 4), depending on the presence and distribution of the components within the FIs, as well as on their (meta-)stability during freezing, which is related to the cooling rate of this operation. Water ice is mainly characterized by an intense, sharp Raman band at ca. 3100 cm^{-1} and by a weak Raman band at ca. 3223 cm^{-1} (refs. 11-13). The position of its main Raman bands shifts depending on temperature, as temperature variations modify water ice volume and thus the internal pressure of the FIs[13]. Methane clathrate is characterized by an intense, sharp Raman band at ca. 2904 cm^{-1} and by a weak Raman band at ca. 2916 cm^{-1} (ref. 14). Hydrohalite is characterized by several Raman bands, whose relative intensities strongly depend on crystal orientation, at ca. 3303, 3325, 3405, 3423, 3436 and 3540 cm^{-1} (refs. 12, 13, 15, 16). Similarly to water ice, also the position of its Raman bands shifts depending on temperature[12]. For some FIs hydrohalite Raman bands are not well-developed, but are replaced by a broad band, possibly indicating a local cryptocrystalline structure of hydrohalite. The 2 phase-bearing FIs with 0 wt.% salinity according to microthermometry and confocal Raman microspectroscopy analyses (sample RP134) do not contain hydrohalite, confirming their NaCl-free composition.

Supplementary Note 3. The Ti and Al concentrations were measured by EMP spot analyses and transects in quartz crystals of 5 samples (Supplementary Data 1, 4). Late-magmatic quartz usually displays higher Ti concentrations than hydrothermal quartz. For late-magmatic quartz (samples RP108, RP116, RP128, RP134 and RP136), crystal cores usually show higher Ti contents than crystal rims (Fig. 5). For hydrothermal quartz (sample RP128), crystals show constant, very low Ti contents from cores to rims. Along with CL images, Ti-Al correlations are fundamental to distinguish between late-magmatic and hydrothermal quartz[17]. Hence, combining EMP data with both colour and grayscale CL images, each quartz generation can be characterized in terms of Ti and Al contents (Supplementary Fig. 5). Late-magmatic quartz is generally characterized by positive correlation of Ti and Al, with a variable range of values for both species, whereas hydrothermal quartz is generally characterized by very low Ti content and highly variable Al content (Supplementary Data 4). Late-magmatic quartz is clearly CL-brighter than hydrothermal quartz and shows CL-bright portions (usually corresponding to crystal cores), displaying Ti content up to some hundreds ppm and Al content up to several hundreds

ppm, CL-dark portions (usually corresponding to crystal mantles or rims), displaying less than 200 ppm for both Ti and Al contents, and rare CL-black portions (as crystal rims), displaying very low values for both Ti and Al contents, usually below detection limit (Supplementary Fig. 5). Hydrothermal quartz is distinctly CL-darker than late-magmatic quartz and shows several CL-brighter and CL-darker growth shells, characterized by very low Ti content, no more than a few tens ppm, and a broad range of Al content, up to a few thousands ppm (Supplementary Fig. 5).

The CL images were collected on FI-bearing quartz crystals of 3 samples (Supplementary Data 1). The CL-intensity of the analysed quartz crystals shows a positive correlation with their Ti concentrations. Hence, late-magmatic quartz is CL-brighter than hydrothermal quartz (Supplementary Fig. 5). Late-magmatic quartz (samples RP116, RP128 and RP136) in CL images usually shows bright purple colour and normal growth zonation (i.e., crystal cores are CL-brighter than crystal rims), with Ti concentration decreasing from core to rims, also in the case of amoeboidal domains with alkali feldspar (Supplementary Fig. 5). Hydrothermal quartz (sample RP128) in CL images usually shows dark purple colour and strong oscillatory growth zonation (i.e., crystals are cyclically CL-brighter and CL-darker along their growth direction), independent from Ti content (constantly very low) but potentially dependent on other trace element variations (Supplementary Fig. 5). Besides the higher transparency of hydrothermal quartz in optical microscopy, the evident lower CL-brightness of hydrothermal quartz compared to late-magmatic quartz is a fundamental character to distinguish between quartz generations on the vein boundaries, where hydrothermal quartz often grew in optical continuity with the previously-crystallized late-magmatic quartz towards the vein centre (Supplementary Fig. 5).

The measured Ti concentrations of quartz (i.e., Ti-in-quartz) were employed to calculate the crystallization temperatures of FI-bearing quartz crystals through geothermobarometry (Supplementary Data 1, 5). The TitaniQ thermobarometry is based on the temperature dependence of Ti^{4+}-Si^{4+} substitution within the quartz lattice, allowing to extrapolate the crystallization temperature of quartz through the measured Ti-in-quartz concentration[18-22]. In the present study, this geothermobarometer, which depends on temperature, pressure and TiO_2 activity relative to rutile saturation ($a_{TiO_2}^{rut.-sat.}$) during quartz crystallization, allowed to constrain the entrapment temperatures of FIs in both late-magmatic and hydrothermal generations of quartz. The crystallization pressure is related to the intrusion depth of CAMP sills within the Paleozoic sequence of the Amazonas Basin, that occurred at about 1-4 km depth[23,24], on average corresponding to ca. 50 MPa. The analysed microgabbros and their hydrothermal veins are not TiO_2-saturated. Ilmenite ($FeTiO_3$) is often associated and in equilibrium with late-magmatic quartz, but rutile (TiO_2) is always absent. Hence, the TiO_2 activity relative to rutile saturation during quartz crystallization is assumed to be 0.6 for both quartz generations, consistently with most silicic melts in magmatic contexts[25].

Using a crystallization pressure of 50 MPa and a TiO_2 activity relative to rutile saturation of 0.6 for both late-magmatic and hydrothermal quartz, we applied the TitaniQ thermobarometry calibration of reference 22, which is reliable especially for low-pressure silica-rich melts. In detail, we used a modified version of their equation 3, in which the Ti-in-quartz concentration is divided by $a_{TiO_2}^{rut.-sat.}$ during crystallization, since the crystallizing system was not TiO_2-saturated[18,25]. The total uncertainty associated with the calculated temperatures is given by the sum of internal and external uncertainties, respectively depending on analytical measurements and on both crystallization pressure and TiO_2 activity relative to rutile saturation. Only Ti concentrations with analytical errors <15 % were used for TitaniQ thermobarometry. An uncertainty of ±10 MPa for the crystallization pressure and of ±0.1 for the TiO_2 activity relative to rutile saturation were considered for each calculated temperature.

The crystallization temperatures of late-magmatic quartz (samples RP108, RP116, RP128, RP134 and RP136) range from 945 to 397 °C, with an average temperature of 662 °C and an average total uncertainty of ±40 °C (Fig. 5; Supplementary Data 5). Calculated temperatures at crystal cores are usually higher than those at crystal rims. The crystallization temperatures of hydrothermal quartz (sample RP128) range from 534 to 416 °C, with an average temperature of 453 °C and an average total uncertainty of ±32 °C (Fig. 5; Supplementary Data 5). The lowest crystallization temperatures estimated for late-magmatic quartz rims (<550 °C) likely indicate low-temperature crystallization, akin to hydrothermal quartz crystals, from Ti-poor fluids circulating along grain boundaries.

Supplementary Figures

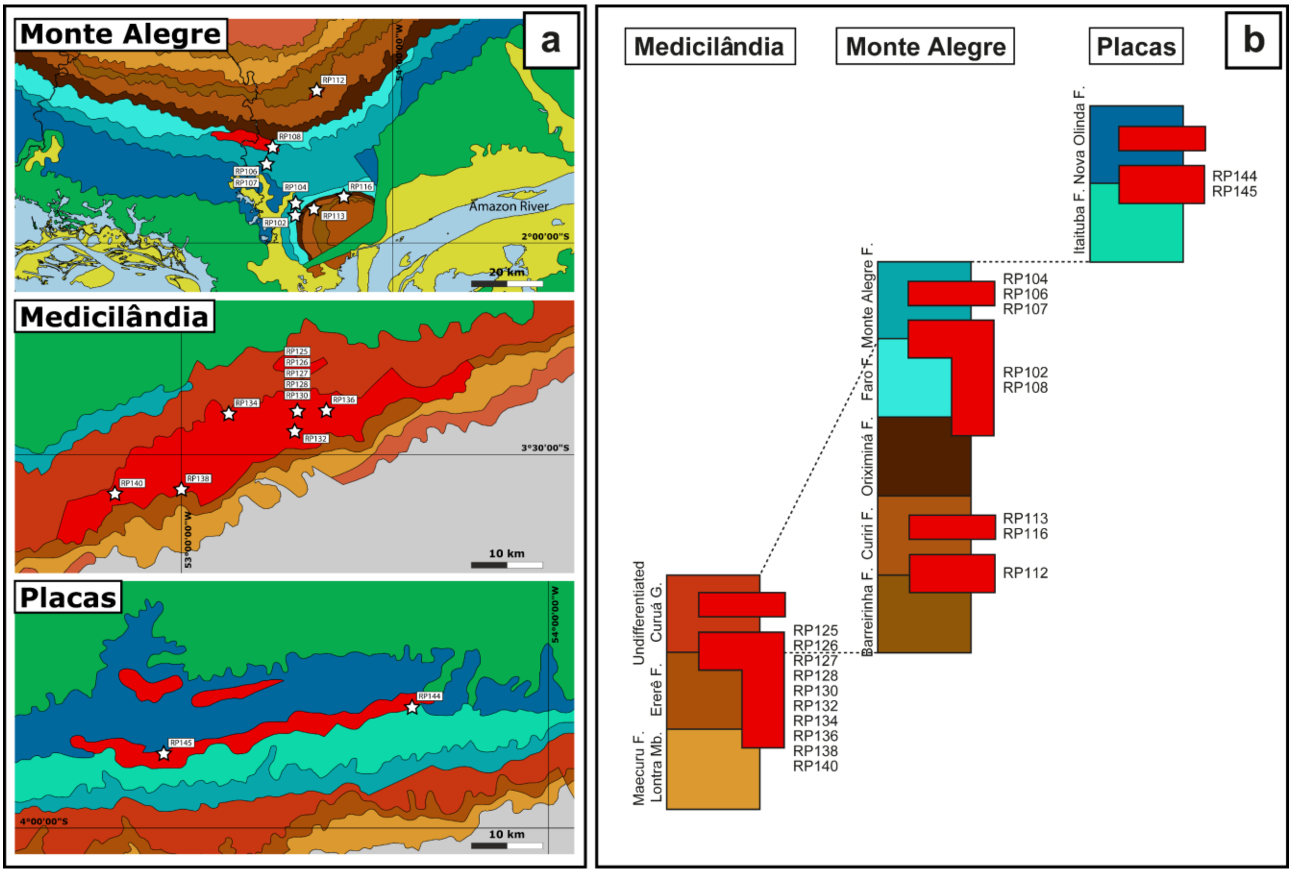

Supplementary Figure 1 Sample provenance and stratigraphic columns. a. The geological maps of Monte Alegre, Medicilândia and Placas (Amazonas Basin) are modified after reference 26. b. The stratigraphic columns schematically represent the position of sills within the sedimentary sequence (thickness not to scale).

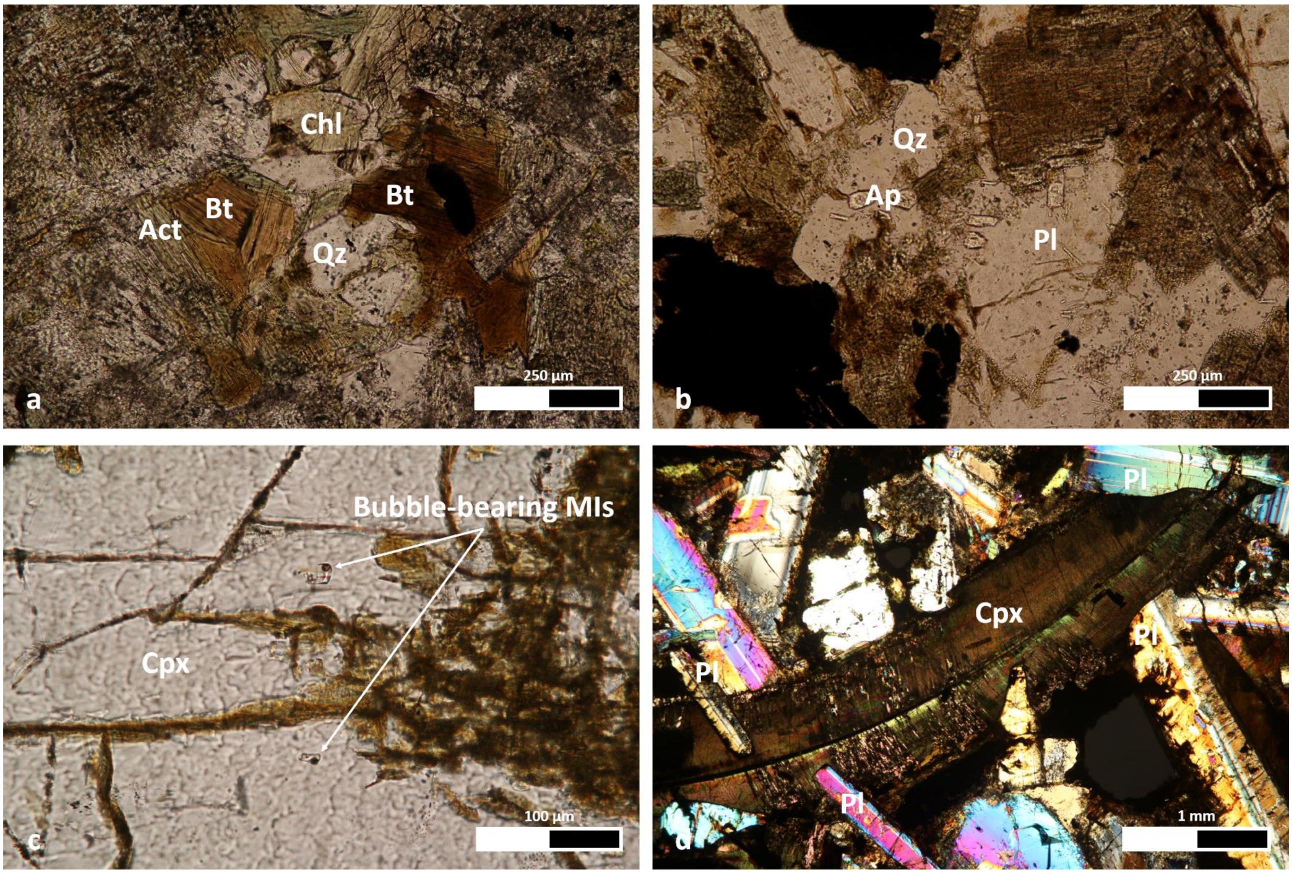

Supplementary Figure 2 Supplementary photomicrographs of microgabbros. a. Interstitial domain composed of late-magmatic quartz (Qz) and red biotite (Bt), partially replaced by hydrothermal actinolite (Act) and chlorite (Chl), forming aggregates of fibrous crystals (sample RP106). b. Tiny crystals of apatite (Ap), associated with interstitial late-magmatic quartz, within the main rock framework composed of early-magmatic plagioclase (Pl) and partially replaced clinopyroxene (sample RP125). c. Bubble-bearing melt inclusions (MIs), hosted in early-magmatic clinopyroxene (Cpx; sample RP145). d. Bent twinned, coarse grained crystal of clinopyroxene, along with early-magmatic plagioclase, showing distinct magmatic viscous-plastic deformation (sample RP134). Transmitted, plane polarized light (a, b, c) and transmitted, crossed polarized light (d).

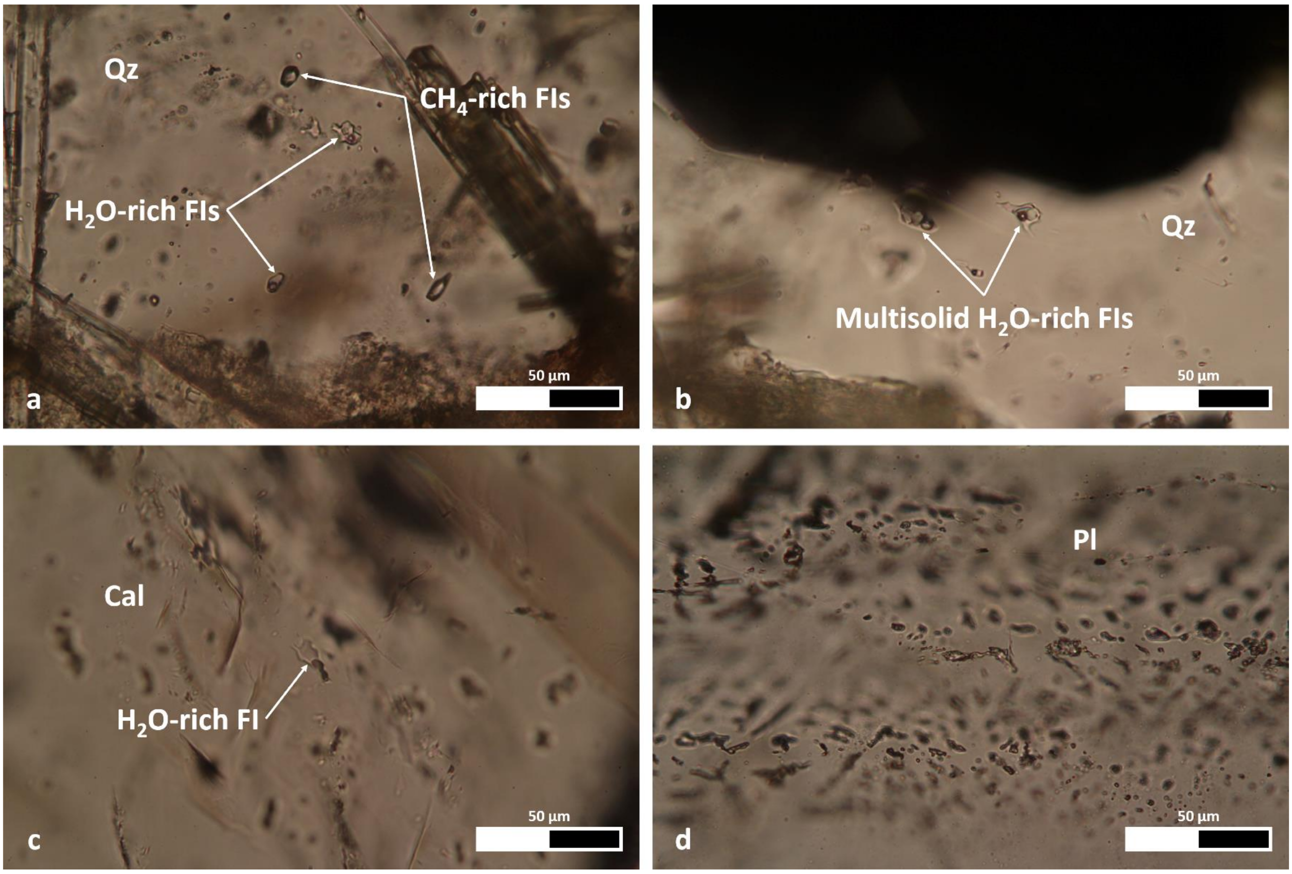

Supplementary Figure 3 Supplementary photomicrographs of FIs. a. H_2O-rich FIs, containing 2 or 3 phases (i.e., liquid + vapour ± solid phases), and CH_4-rich FIs, containing 2 phases (i.e., liquid + vapour phases), hosted in late-magmatic quartz (Qz; sample RP116). b. Multi-solid H_2O-rich FIs, containing more than 1 solid phase besides vapour and liquid phases, hosted in late-magmatic quartz (sample RP125). c. H_2O-rich FI, containing 2 phases (i.e., liquid + vapour phases), hosted in hydrothermal calcite (Cal; sample RP128). d. Secondary inclusions (not analysed) aligned along sealed fracture planes, hosted in magmatic plagioclase (Pl; sample RP116). Transmitted, plane polarized light.

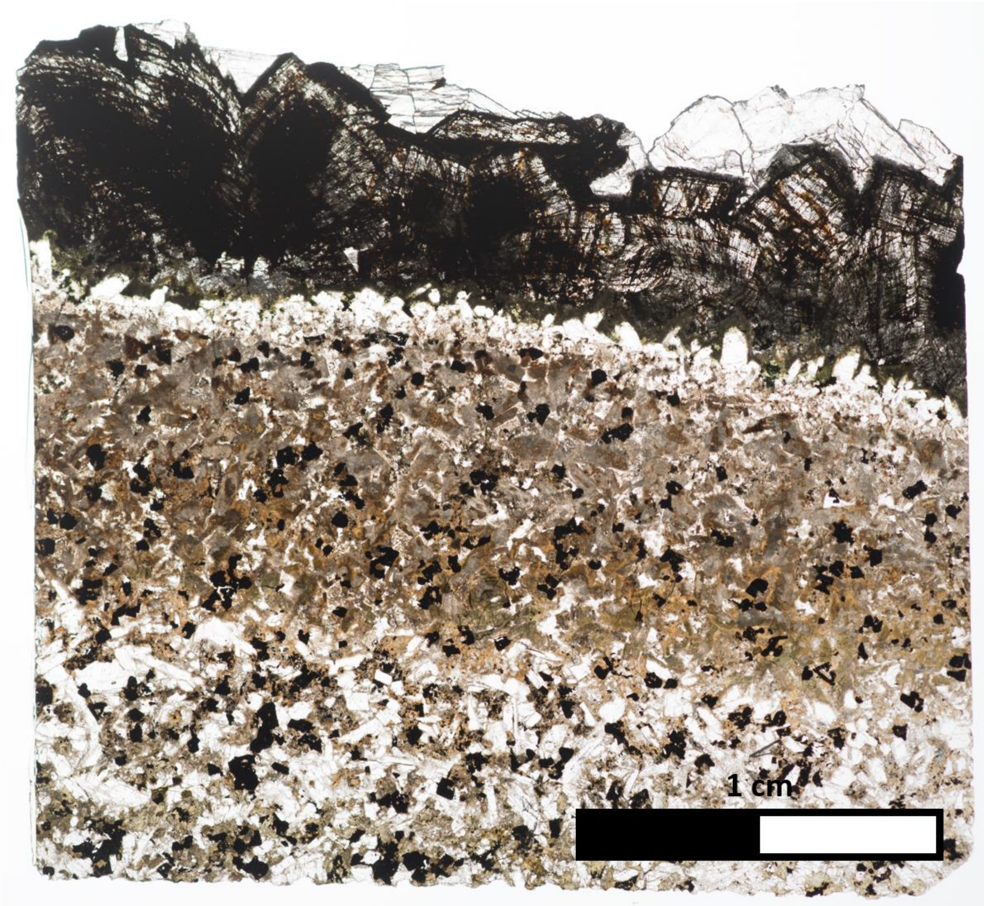

Supplementary Figure 4 Photograph of hydrothermal vein crosscutting the microgabbro in sample RP128. Both the macroscopically unaltered magmatic rock (lowermost part in the picture) and the alteration band surrounding the hydrothermal vein (uppermost part in the picture) display hydrothermal alteration. Within the alteration band only quartz is preserved. From rim to centre of the symmetrical hydrothermal vein, there are a thin level of transparent quartz (at hydrothermal vein rims), a thin level of very fine grained chlorite, sericite and pyrrhotite, a thick level of turbid Ca-Mg-Fe carbonates, such as siderite, ankerite and possibly dolomite, and a thin level of transparent calcite (at hydrothermal vein centre). The symmetrical structure of this hydrothermal vein reflects the precipitation order of the hydrothermal minerals, according to the compositional evolution of the hydrothermal system (i.e., from silicate to carbonate system). Within the hydrothermal vein, 2 or 3 phase-bearing FIs (i.e., liquid + vapour ± solid phases) are present in both quartz and calcite. See also Supplementary Figure 5.

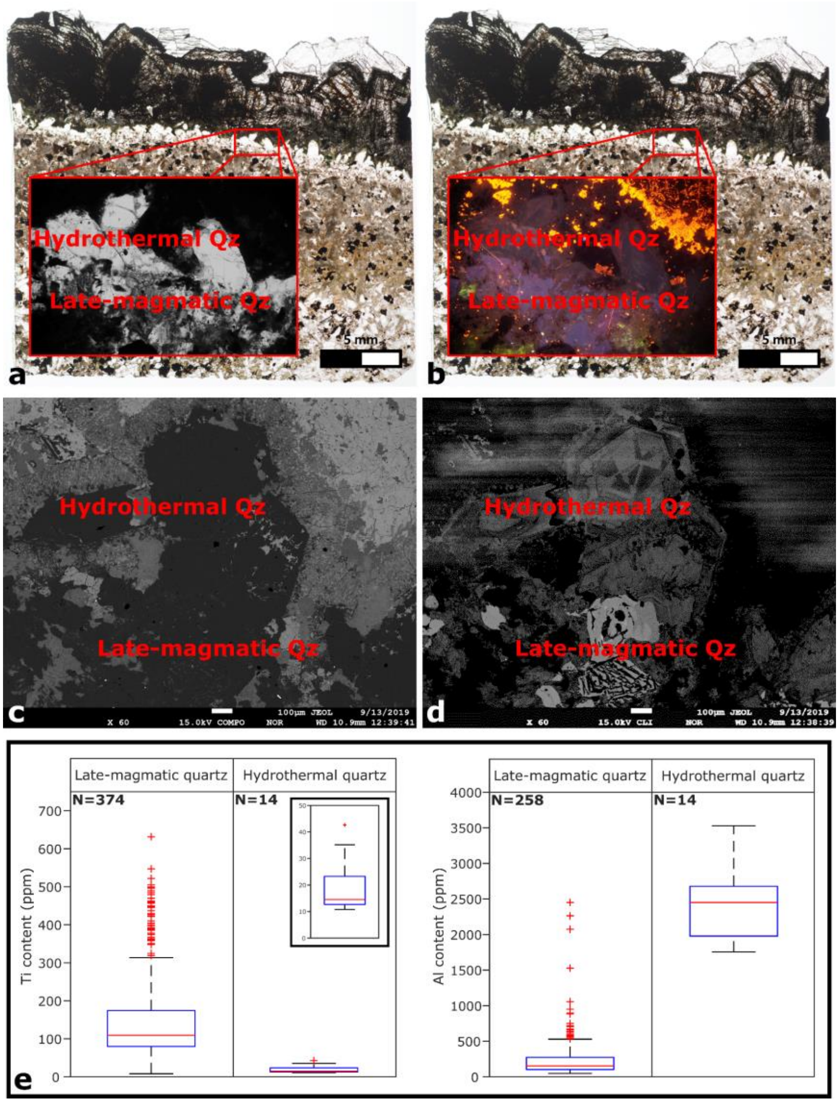

Supplementary Figure 5 Cathodoluminescence images along with Ti and Al concentrations of both late-magmatic and hydrothermal quartz. a, b. Photograph of the hydrothermal vein (sample RP128), displaying an enlargement of the vein boundary with an optical photomicrograph (a) and the corresponding colour CL image (b). In this site late-magmatic and hydrothermal quartz (Qz) crystals are present. c, d. Backscattered-electron (BSE) and grayscale CL images for a similar site within the same sample. The late-magmatic quartz crystals, hosting CH_4-bearing FIs, are typically CL-brighter than the hydrothermal ones, hosting CH_4-free FIs. e. Ti and Al concentrations of late-magmatic and hydrothermal quartz in box-and-whisker plots. In box-and-whisker plots, the middle line of the box indicates the median, the bottom and top edges of the box show the lower and upper quartiles respectively, the whiskers extend to the minimum and maximum data that are not outliers, and the outliers are plotted individually. These data are reported in Supplementary Data 4. Note that the plotted data are the same plotted in Figure 5 for quartz crystallization temperatures, excluding those displaying below-detection-limit values or >15 % analytical errors for Al.

Supplementary References

[1] Capriolo, M. *et al.* Deep CO_2 in the end-Triassic Central Atlantic Magmatic Province. *Nat. Commun.* **11**, 1670 (2020).

[2] Frezzotti, M. L., Tecce, F. & Casagli, A. Raman spectroscopy for fluid inclusion analysis. *J. Geochem. Explor.* **112**, 1-20 (2012).

[3] Brunsgaard Hansen, S. & Berg, R. W. Raman Spectroscopic Studies of Methane Gas Hydrates. *Appl. Spectrosc. Rev.* **44**, 168-179 (2009).

[4] Guillaume, D., Teinturier, S., Dubessy, J. & Pironon, J. Calibration of methane analysis by Raman spectroscopy in H_2O-NaCl-CH_4 fluid inclusions. *Chem. Geol.* **194**, 41-49 (2003).

[5] Du, Z. *et al.* In situ Raman spectroscopy study of synthetic gas hydrate formed by cold seep flow in the South China Sea. *J. Asian Earth Sci.* **168**, 197-206 (2018).

[6] Bodnar, R. J. & Frezzotti, M. L. Microscale Chemistry: Raman Analysis of Fluid and Melt Inclusions. *Elements* **16**, 93-98 (2020).

[7] Lu, W., Chou, I-M., Burruss, R. C. & Song, Y. A unified equation for calculating methane vapor pressures in the CH_4-H_2O system with measured Raman shifts. *Geochim. Cosmochim. Acta* **71**, 3969-3978 (2007).

[8] Shang, L., Chou, I-M., Burruss, R. C., Hu, R. & Bi, X. Raman spectroscopic characterization of CH_4 density over a wide range of temperature and pressure. *J. Raman Spectrosc.* **45**, 696-702 (2014).

[9] Lin, F., Sum, A. K. & Bodnar, R. J. Correlation of methane Raman ν_1 band position with fluid density and interactions at the molecular level. *J. Raman Spectrosc.* **38**, 1510-1515 (2007).

[10] Cohen-Adad, R. & Lorimer, J. W., Eds. *Alkali Metal and Ammonium Chlorides in Water and Heavy Water (Binary Systems)* (vol. 47 of *Solubility Data Series*, Pergamon Press, Oxford, UK, 1991).

[11] Fukazawa, H. & Mae, S. "The vibrational spectra of ice Ih and polar ice" in *Physics of Ice Core Records*, Hondoh, T., Ed. (Hokkaido University Press, Sapporo, Japan, 2000) pp. 25-42.

[12] Bakker, R. J. Raman spectra of fluid and crystal mixtures in the systems H_2O, H_2O-NaCl and H_2O-$MgCl_2$ at low temperatures: Applications to fluid-inclusion research. *Can. Mineral.* **42**, 1283-1314 (2004).

[13] Baumgartner, M. & Bakker, R. J. Raman spectra of ice and salt hydrates in synthetic fluid inclusions. *Chem. Geol.* **275**, 58-66 (2010).

[14] Sum, A. K., Burruss, R. C. & Sloan Jr., E. D. Measurement of Clathrate Hydrates via Raman Spectroscopy. *J. Phys. Chem. B* **101**, 7371-7377 (1997).

[15] Dubessy, J., Audeoud, D., Wilkins, R. & Kosztolanyi, C. The use of the Raman microprobe mole in the determination of the electrolytes dissolved in the aqueous phase of fluid inclusions. *Chem. Geol.* **37**, 137-150 (1982).

[16] Ni, P., Ding, J. & Rao, B. In situ cryogenic Raman spectroscopic studies on the synthetic fluid inclusions in the systems H_2O and NaCl-H_2O. *Chi. Sci. Bull.* **51**, 108-114 (2006).

[17] Ackerson, M. R., Tailby, N. D. & Watson, E. B. Trace elements in quartz shed light on sediment provenance. *Geochem. Geophy. Geosy.* **16**, 1894-1904 (2015).

[18] Wark, D. A. & Watson, E. B. TitaniQ: a titanium-in-quartz geothermometer. *Contrib. Mineral. Petrol.* **152**, 743-754 (2006).

[19] Thomas, J. B. *et al.* TitaniQ under pressure: the effect of pressure and temperature on the solubility of Ti in quartz. *Contrib. Mineral. Petrol.* **160**, 743-759 (2010).

[20] Huang, R. & Audétat, A. The titanium-in-quartz (TitaniQ) thermobarometer: A critical examination and re-calibration. *Geochim. Cosmochim. Acta* **84**, 75-89 (2012).

[21] Thomas, J. B., Watson, E. B., Spear, F. S. & Wark, D. A. TitaniQ recrystallized: experimental confirmation of the original Ti-in-quartz calibrations. *Contrib. Mineral. Petrol.* **169 (27)**, 1-16 (2015).

[22] Zhang, C. *et al.* Ti-in-quartz thermobarometry and TiO_2 solubility in rhyolitic melts: new experiments and parametrization. *Earth Planet. Sci. Lett.* **538**, 116213 (2020).

[23] Gonzaga, F. G., Gonçalves, F. T. T. & Coutinho, L. F. C. "Petroleum geology of the Amazonas Basin, Brazil: modeling of hydrocarbon generation and migration" in *Petroleum systems of South Atlantic margins*, Mello, M. R. & Katz, B. J., Eds. (AAPG Memoir, 2000) vol. 73, chap. 13.

[24] Caputo, M. V. & Soares, E. A. A. Eustatic and tectonic change effects in the reversion of the transcontinental Amazon River drainage system. *Brazilian J. Geol.* **46 (2)**, 301-328 (2016).

[25] Hayden, L. A. & Watson, E. B. Rutile saturation in hydrous siliceous melts and its bearing on Ti-thermometry of quartz and zircon. *Earth Planet. Sci. Lett.* **258**, 561-568 (2007).

[26] Vasquez, M. L., Sousa, C. S. & Carvalho, J. M. A. Mapa Geológico do Estado do Pará (1:1.000.000). Programa Geologia do Brasil (2008).

[27] Davies, J. H. F. L. *et al.* End-Triassic mass extinction started by intrusive CAMP activity. *Nat. Commun.* **8**, 15596 (2017).

[28] Davies, J. H. F. L. *et al.* Zircon petrochronology in large igneous provinces reveals upper crustal contamination processes: new U-Pb ages, Hf and O isotopes, and trace elements from the Central Atlantic magmatic province (CAMP). *Contrib. Mineral. Petrol.* **176**, 9 (2021).

[29] Marzoli, A. *et al.* The Central Atlantic Magmatic Province (CAMP) in Morocco. *J. Petrol.* **60 (5)**, 945-996 (2019).

[30] Merle, R. *et al.* $^{40}Ar/^{39}Ar$ ages and Sr-Nd-Pb-Os geochemistry of CAMP tholeiites from Western Maranhão basin (NE Brazil). *Lithos* **122**, 137-151 (2011).

[31] Ptáček, M. P., Dauphas, N. & Greber, N. D. Chemical evolution of the continental crust from a data-driven inversion of terrigenous sediment compositions. *Earth Planet. Sci. Lett.* **539**, 116090 (2020).
