# Supplementary material for: Massive methane fluxing from magma–sediment interaction in the end-Triassic Central Atlantic Magmatic Province
Source: Nat Commun. 2021 Sep 20;12:5534. doi: 10.1038/s41467-021-25510-w (PMC8452664; doi:10.1038/s41467-021-25510-w)
Supplement: Supplementary file 2 — Supplementary Information [file 41467_2021_25510_MOESM2_ESM.pdf]

Description of Supplementary and Source Data files

Massive methane fluxing from magma-sediment interaction in the end-Triassic Central Atlantic Magmatic Province

Capriolo et al.

Supplementary Data

Supplementary Data 1 Dataset for all selected samples. The crosses indicate which analytical techniques were applied for each investigated sample.

Supplementary Data 2 XRF data of whole-rock major element composition for the most representative FI-bearing samples. The normative contents of quartz (Qz), hypersthene (Hy) and olivine (Ol), calculated using the CIPW norm, are also shown for these samples. The chemical composition of CAMP basalts (samples AN133 and M13) and assimilated shale (sample Shale) used in Rhyolite-MELTS modelling is reported.

Supplementary Data 3 Raman band positions of CH_4, calibrated with neon emission lines, and density of CH_4 within liquid- and vapour-rich FIs, calculated using the densimeters of references 7 and 8, for samples RP106, RP116, RP128 and RP134. All data were acquired at ambient temperature.

Supplementary Data 4 EMP data of Ti and Al concentrations in quartz and relative analytical errors for samples RP108, RP116, RP128, RP134 and RP136. Cells marked by *b.d.l.* indicate below-detection-limit values. Data with analytical errors >15 % were considered as not reliable and were not considered further. Data with Al content >5000 ppm were considered as not on pure quartz and were not considered further for both Ti and Al. Potential contamination and secondary fluorescence may have occurred from adjacent mineral phases (e.g., from alkali feldspar when measuring sites in correspondence of those interstitial micrographic intergrowths of quartz and alkali feldspar). In both Ti and Al datasets, spurious data are possibly due to secondary fluorescence or to cracks in the analysed crystals.

Supplementary Data 5 Ti-in-quartz concentrations, relative analytical errors and calculated crystallization temperatures with uncertainties, determined using the TitaniQ thermobarometry calibration of reference 22, for samples RP108, RP116, RP128, RP134 and RP136. Minimum, maximum and average temperatures and uncertainties are indicated for each sample, and average temperatures and uncertainties are indicated for each quartz generation. Data on hydrothermal quartz are highlighted in light blue.

Supplementary Data 6 Rhyolite-MELTS modelling outputs for both low- and high-Ti basalts. For low-Ti basalts, quartz saturation was calculated to occur at ca. 790 °C and 50 MPa without assimilation, and at ca. 800 °C and 50 MPa with 10 wt.% assimilation. At the solidus (ca. 780 °C and 50 MPa) the amount of crystallized quartz is about 3 wt.% without assimilation, and about 5 wt.% with 10 wt.% assimilation. For high Ti basalts, quartz saturation does not occur without assimilation, but it was calculated to occur at ca. 810 °C and 50 MPa, and at ca. 760 °C and 100 MPa with 20 wt.% assimilation. At the solidus (ca. 750 °C and 100 MPa) the amount of crystallized quartz is about 4 wt.% with 20 wt.% assimilation.

Source Data

Source Data 1 Confocal Raman microspectroscopy data.

Source Data 2 Confocal Raman microspectroscopy combined with microthermometry data.
